# New potential antitumoral fluorescent tetracyclic thieno[3,2-*b*]pyridine derivatives: interaction with DNA and nanosized liposomes

**DOI:** 10.1186/1556-276X-6-379

**Published:** 2011-05-12

**Authors:** Elisabete MS Castanheira, Maria Solange D Carvalho, Ana Rita O Rodrigues, Ricardo C Calhelha, Maria-João RP Queiroz

**Affiliations:** 1 Centre of Physics (CFUM), University of Minho, Campus de Gualtar, Braga, 4710-057, Portugal; 2 Centre of Chemistry (CQ-UM), University of Minho, Campus de Gualtar, Braga, 4710-057, Portugal

## Abstract

Fluorescence properties of two new potential antitumoral tetracyclic thieno[3,2-*b*]pyridine derivatives were studied in solution and in liposomes of DPPC (dipalmitoyl phosphatidylcholine), egg lecithin (phosphatidylcholine from egg yolk; Egg-PC) and DODAB (dioctadecyldimethylammonium bromide). Compound **1**, pyrido[2',3':3,2]thieno[4,5-*d*]pyrido[1,2-*a*]pyrimidin-6-one, exhibits reasonably high fluorescence quantum yields in all solvents studied (0.20 ≤ Φ_F _≤ 0.30), while for compound **2**, 3-[(*p*-methoxyphenyl)ethynyl]pyrido[2',3':3,2]thieno[4,5-*d*]pyrido[1,2-*a*]pyrimidin-6-one, the values are much lower (0.01 ≤ Φ_F _≤ 0.05). The interaction of these compounds with salmon sperm DNA was studied using spectroscopic methods, allowing the determination of intrinsic binding constants, *K*_i _= (8.7 ± 0.9) × 10^3 ^M^-1 ^for compound **1 **and *K*_i _= (5.9 ± 0.6) × 10^3 ^M^-1 ^for **2**, and binding site sizes of *n *= 11 ± 3 and *n *= 7 ± 2 base pairs, respectively. Compound **2 **is the most intercalative compound in salmon sperm DNA (35%), while for compound **1 **only 11% of the molecules are intercalated. Studies of incorporation of both compounds in liposomes of DPPC, Egg-PC and DODAB revealed that compound **2 **is mainly located in the hydrophobic region of the lipid bilayer, while compound **1 **prefers a hydrated and fluid environment.

## Introduction

Liposomes are among technological delivery developments for chemotherapeutic drugs in the treatment of cancer. This technique can potentially overcome many common pharmacologic problems, such as those involving solubility, pharmacokinetics, in vivo stability and toxicity [1-3]. Liposomes are closed spherical vesicles consisting of a lipid bilayer that encapsulates an aqueous phase in which hydrophilic drugs can be stored, while water insoluble compounds can be incorporated in the hydrophobic region of the lipid bilayer [4].

**Figure 1:**
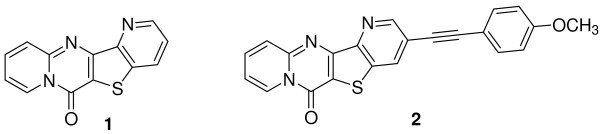
**Structure of the compounds 1 and 2**.

In this work, two new potential antitumoral fluorescent planar tetracyclic thieno[3,2-*b*]pyridine derivatives **1 **and **2 **(Figure 1), previously synthesized by some of us [5], were encapsulated in liposomes of DPPC (dipalmitoyl phosphatidylcholine), egg lecithin (phosphatidylcholine from egg yolk) and DODAB (dioctadecyldimethylammonium bromide). DPPC and egg lecithin [egg yolk phosphatidylcholine (Egg-PC)] are neutral components of biological membranes, while cationic liposomes based on the synthetic lipid DODAB have been used as vehicles for DNA transfection and drug delivery [6]. These studies are important keeping in mind future drug delivery applications using these compounds as anticancer drugs.

Due to the antitumoral potential of the two compounds **1 **and **2**, related with their possible intercalation between the DNA base pairs, interactions with natural double-stranded salmon sperm DNA were studied. These interactions can be assessed using spectroscopic measurements, which are important tools for monitoring DNA-binding processes. The investigation based on DNA interactions has a key importance in order to understand the mechanisms of action of antitumor and antiviral drugs and to design new DNA-targeted drugs [7,8]. Small molecules are stabilized on groove binding and intercalation with DNA through a series of associative interactions such as π-stacking, hydrogen bonding, attractive van der Waals and hydrophobic interactions [8]. The occurrence of intercalation seems to be an essential (but not sufficient) step for antitumoral activity [7]. Fluorescence quenching experiments using external quenchers are also very useful to distinguish between DNA binding modes [9] since intercalated molecules are less accessible to anionic quenchers due to electrostatic repulsion with negatively charged DNA [10].

## Experimental

Salmon sperm DNA from Invitrogen (Carlsbad, CA, USA) and compounds stock solutions were prepared in 10 mM Tris-HCl buffer (pH = 7.4), with 1 mM EDTA. The DNA concentration in number of bases was determined from the molar absorption coefficient, ε = 6600 M^-1 ^cm^-1 ^at 260 nm [11]. Fluorescence spectra of several solutions with different [DNA]/[compound] ratios and constant compound concentration (5 × 10^-6 ^M) were recorded. The solutions were left several hours to stabilize.

Dipalmitoyl phosphatidylcholine (DPPC), egg yolk phosphatidylcholine (Egg-PC), from Sigma-Aldrich (St. Louis, Missouri, USA), and dioctadecyldimethylammonium bromide (DODAB), from Tokyo Kasei (Tokyo, Japan), were used as received. Liposomes were prepared by the ethanolic injection method, previously used for the preparation of Egg-PC and DPPC liposomes [12-15] and DODAB vesicles [16,17]. An ethanolic solution of a lipid/compound mixture was injected in an aqueous buffer solution under vigorous stirring, above the melting transition temperature of the lipid (approx. 41°C for DPPC [18] and 45°C for DODAB [19]). The final lipid concentration was 1 mM, with a compound/lipid molar ratio of 1:500. One millilitre solutions of liposome dispersions were placed in 3 mL disposable polystyrene cuvettes for dynamic light scattering (DLS) measurements in a Malvern ZetaSizer Nano ZS particle analyzer (Worcestershire, UK). Five independent measurements were performed for each sample. Malvern Dispersion Technology Software (DTS) (Worcestershire, UK) was used with multiple narrow mode (high resolution) data processing, and mean size (nm) and error values were considered.

Absorption spectra were recorded in a Shimadzu UV-3101PC UV-Vis-NIR spectrophotometer (Kyoto, Japan) and fluorescence measurements were obtained in a Fluorolog 3 spectrofluorimeter (HORIBA Scientific, Kyoto, Japan) equipped with Glan-Thompson polarizers. Fluorescence spectra were corrected for the instrumental response of the system. The fluorescence quantum yields were determined by the standard method [20,21], using 9,10-diphenylanthracene in ethanol as reference, Φ_r _= 0.95 [22]. The solutions were previously bubbled for 20 min with ultrapure nitrogen.

## Results and discussion

**Figure 2:**
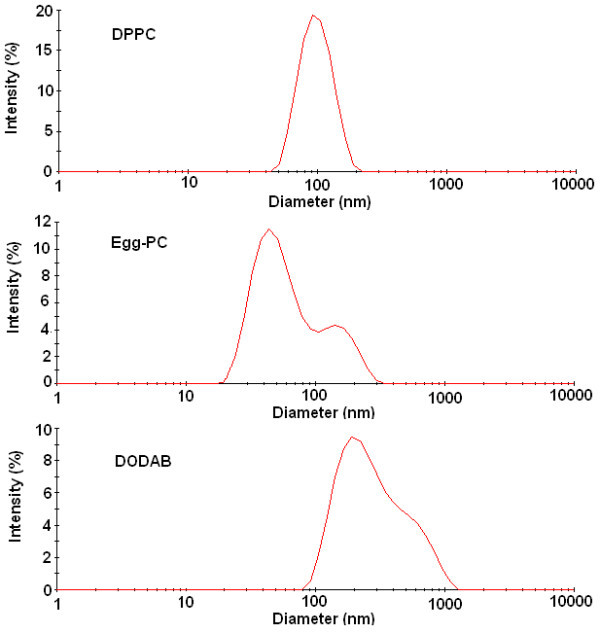
**Size distributions obtained by dynamic light scattering (DLS) for DPPC, Egg-PC and DODAB liposomes prepared by the ethanolic injection method**.

The size and size distribution of the liposomes prepared was obtained by DLS. All the liposomes have a mean hydrodynamic radius lower than 150 nm and generally low polydispersity. For Egg-PC and DODAB liposomes, the size distributions are bimodals and broader than for DPPC liposomes, the Egg-PC being the more polydisperse (Figure 2). The ethanolic injection method was described to produce phospholipid small unilamellar vesicles (SV) [12-15]. Accordingly, DPPC and Egg-PC liposomes obtained here are in this category, with a mean diameter of around 90 nm for DPPC and 50 nm for Egg-PC. DODAB liposomes exhibit a significantly larger mean diameter (around 270 nm) than the phospholipid ones. The size of DODAB vesicles strongly depends on the preparation method, sonication and ethanolic injection giving small DODAB vesicles [17,23,24], while injection using chloroform yielded large DODAB vesicles [16]. Besides, spontaneously prepared DODAB liposomes have a much larger size (hydrodynamic radius around 337 nm [25]), being considered giant unilamellar vesicles (GUV). The DODAB liposomes mean diameter obtained here (*ca*. 270 nm) compares well with the reported value of 249 nm for DODAB SV [16]. In all samples, no experimental evidence of the presence of open bilayer fragments (diameter lower than 10 nm [17]) was obtained (Figure 2).

**Figure 3:**
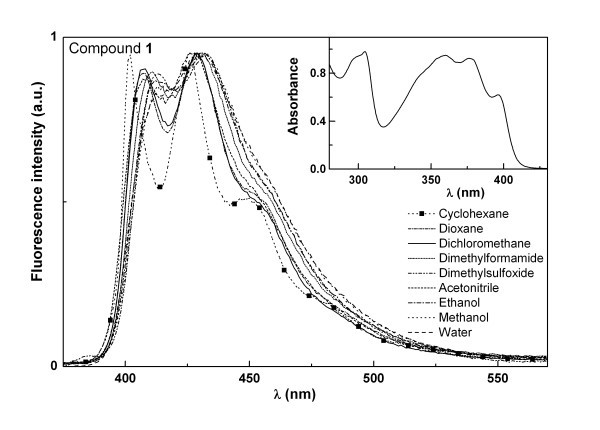
**Normalized fluorescence spectra (λ_exc _= 360 nm) of compound 1 (4 × 10^-6 ^M) in several solvents; the inset shows the absorption spectrum of 1 in dichloromethane (1 × 10^-4 ^M) as an example**.

**Figure 4:**
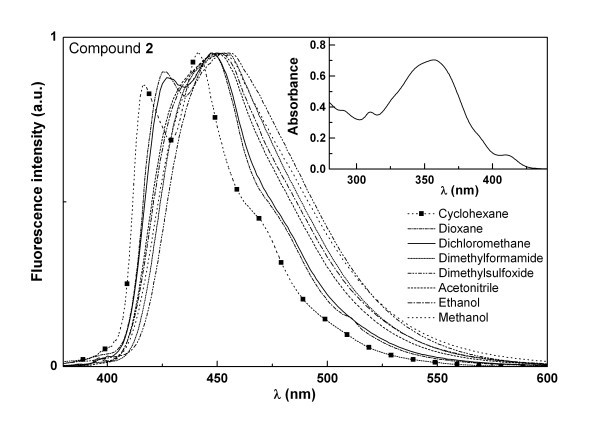
**Normalized fluorescence spectra (λ_exc _= 360 nm) of compound 2 (4 × 10^-6 ^M) in several solvents; the inset shows the absorption spectrum of 2 in dichloromethane (2 × 10^-5 ^M) as an example**.

The absorption and fluorescence properties of compounds **1 **and **2 **were studied in several solvents (Table 1). The normalized fluorescence spectra of compounds **1 **and **2 **are shown in Figures 3 and 4. The fluorescence emission maximum of both compounds displays a loss of vibrational structure in polar solvents together with a small red shift (Figures 3 and 4), indicating some charge transfer character of the excited state [26]. The red shifts are more significant for compound **2 **(Table 1), which may be due to a higher capability of this compound to establish hydrogen bonds with protic solvents (especially with water), due to the presence of the OCH_3 _group. Compound **1 **has significantly higher fluorescence quantum yields (between 20 and 30%) than compound **2 **(Φ_F _between 1 and 5%), showing that the functionalization of the pyridine ring with a triple bond linked to a *p*-methoxyphenyl group causes a significant enhance of the non-radiative deactivation pathways. The fluorescence quantum yields of compound **1 **are also higher than the ones of a benzo[*b*]thiophene derivative of the same type, a benzothienopyridopyrimidone [27], in which the benzene ring linked to the thiophene is substituted in compound **1 **by a pyridine ring. The intrinsic fluorescence of compounds **1 **and **2 **can be used to monitor interactions with DNA and compounds behaviour when encapsulated in liposomes.

**Table 1:** Maximum absorption (λ_abs_) and emission (λ_em_) wavelengths, molar absorption coefficients (ε) and fluorescence quantum yields of compounds 1 and 2 in several solvents

Solvent	λ_abs _(nm) (ε/10^4 ^M^-1 ^cm^-1^)		λ_em _(nm)		Φ_F_	
	1	2	1	2	1	2
Cyclohexane	398 (0.84); 377 (1.24); 360 (1.27); 305 (0.95); 258 (3.93)	411 *sh *(0.33); 354 (2.19); 347 (2.37); 308 (1.25); 291 (1.12); 270 (1.40)	402; 426; 452 *sh*	417; 441	0.20	0.047
Dioxane	398 (0.76); 377 (1.18); 359 (1.20); 305 (1.17); 258 (3.60)	411 *sh *(0.66); 356 (5.36); 346 (5.40); 309 (3.23); 291 (2.98); 272 (3.33)	407; 428; 455 *sh*	425; 449	0.29	0.054
Dichloromethane	397 (0.58); 377 (0.91); 360 (0.93); 305 (0.97); 259 (2.70)	410 *sh *(0.55); 357 (4.37); 311 (2.28); 290 (2.29); 273 (2.78)	408; 429	427; 448	0.26	0.022
Acetonitrile	395 (0.68); 376 (1.06); 358 (1.06); 304 (1.09); 256 (3.32)	409 *sh *(0.66); 355 (5.76); 308 (3.41); 289 (3.20); 271 (3.67)	408; 428	450	0.21	0.036
*N,N*-Dimethylformamide^a^	397 (0.78); 377 (1.19); 360 (1.16); 305 (1.19)	411 *sh *(0.69); 356 (5.52); 311 (3.11); 290 (2.86)	411; 430	453	0.30	0.047
Dimethylsulfoxide^a^	397 (0.77); 378 (1.17); 361 (1.14); 305 (1.17)	412 *sh *(0.61); 357 (4.70); 313 (2.52)	413; 432	455	0.28	0.048
Ethanol	396 (0.69); 375 (1.13); 358 (1.17); 304 (1.40); 256 (3.59)	408 *sh *(0.72); 355 (5.50); 311 (2.95); 272 (3.69)	412; 431	452	0.27	0.041
Methanol	395 (0.67); 374 (1.08); 358 (1.10); 304 (1.34); 256 (3.43)	408 *sh *(0.62); 354 (5.00); 311 (2.80); 272 (3.41)	413; 433	453	0.26	0.040
Water	394 (0.41); 374 (0.57); 361 (0.58); 303 (0.93); 256 (2.07)	420 *sh *(0.26); 358 (0.87); 314 (0.94); 278 (0.97)	413 *sh*; 433	505	0.22	0.012

^a^Solvent cut-offs: *N,N*-Dimethylformamide: 275 nm; Dimethylsulfoxide: 280 nm; *sh*: shoulder.

**Figure 5:**
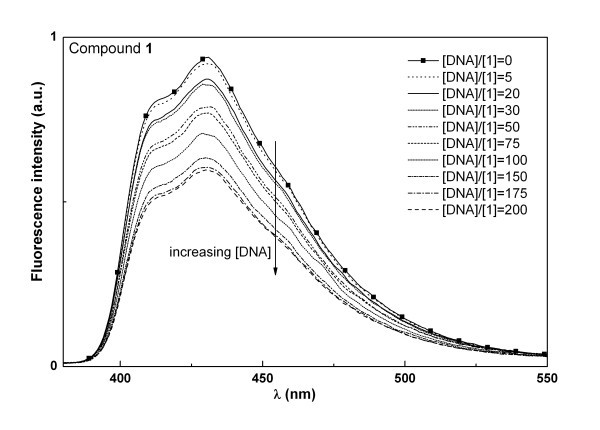
**Fluorescence spectra of compound 1 (5 × 10^-6 ^M) in 0.01 M Tris-HCl buffer (pH = 7.2), with increasing DNA content**.

**Figure 6:**
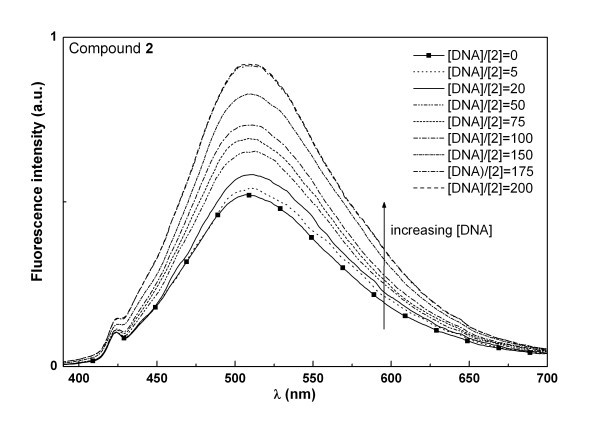
**Fluorescence spectra of compound 2 (5 × 10^-6 ^M) in 0.01 M Tris-HCl buffer (pH = 7.2), with increasing DNA content**.

Both compounds **1 **and **2 **were tested for their interaction with natural salmon sperm DNA using spectroscopic methods. For compound **1**, fluorescence intensity decreases with increasing DNA concentration, while the opposite happens for compound **2 **(Figures 5 and 6). This behaviour, also previously observed for differently substituted tetracyclic lactams [28], may indicate a different type of interaction of both compounds with the DNA molecule. For the two compounds, full saturation (corresponding to spectral invariance with increasing DNA concentration) is attained at [DNA]/[compound] = 200, meaning that total binding is achieved at this ratio. The high [DNA]/[compound] ratio needed for total binding, together with the negligible changes observed in absorption spectra (not shown), point to a weak interaction of these molecules with the nucleic acid.

The intrinsic binding constants (*K*_i_) and binding site sizes (*n*) were determined (Table 2) through the McGhee and von Hippel modification of Scatchard plot (Equation 1) [29],(1)

**Table 2:** Values of the intrinsic binding constants (*K*_i_) and binding site sizes (*n*) and fraction of compound molecules accessible to external quenchers (*f*_*a*_) for interaction with salmon sperm DNA

Compound	***K***_**i **_**(M**^-**1**^**)**	*n*	***f***_**a**_
**1**	(8.7 ± 0.9) × 10^3^	11 ± 3	0.89
**2**	(5.9 ± 0.6) × 10^3^	7 ± 2	0.65

where *K*_i _is the intrinsic binding constant, *n *the binding site size, *r *the ratio *c*_b_/[DNA] and *c*_b _and *c*_f _the concentrations of bound and free compound, respectively, calculated by(2)

being *I*_F,0 _the fluorescence intensity of the free compound and *I*_F,b _the fluorescence intensity of the bound compound at total binding. The binding constants (Table 2) are moderately low, with a large number of base pairs between consecutive intercalated compound molecules (*n*).

**Figure 7:**
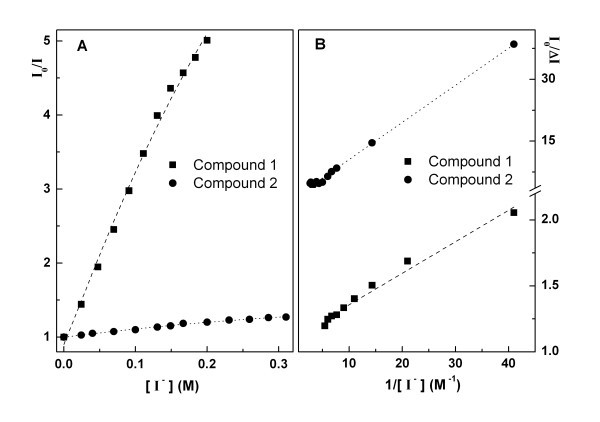
**Stern-Volmer plots for quenching with iodide ion of compounds 1 and 2 for [DNA]/[compound] = 200 (A) and corresponding modified Stern-Volmer plots (B)**.

Anionic quenchers can be useful in distinguishing between DNA binding modes [9,10]. Compounds that are bound at the DNA surface (groove binding or electrostatic binding) are more accessible and emission from these molecules can be quenched more efficiently. Fluorescence quenching measurements using iodide ion showed that the usual Stern-Volmer plots (plots of the fluorescence intensity ratio in the absence, *I*_0_, and presence, *I*, of quencher *vs*. quencher concentration) are not linear and exhibit a downward curvature (Figure 7A). This indicates that some compound molecules are not accessible to the anionic quencher, being intercalated between DNA base pairs. The modified Stern-Volmer plot [30] (Equation 3) allows the determination of the fraction of compound molecules accessible to quencher,(3)

where *I*_0 _is the fluorescence intensity in the absence of quencher, Δ*I *= *I*_0 _- *I*, *K*_SV _the Stern-Volmer constant, [Q] the quencher concentration and *f*_a _the fraction of molecules accessible to quencher.

The representations of the modified Stern-Volmer plot are reasonably linear (Figure 7B) and the *f*_a _values are in Table 2. Both compounds exhibit some intercalation in DNA, compound **2 **being the more intercalative one, with a lower fraction (65%) of molecules accessible to anionic quencher. The higher hydrophobic character of compound **2**, promoted by the functionalization of the pyridine with a triple bond linked to a *p*-methoxyphenyl group, may justify this behaviour. As both compounds **1 **and **2 **are neutral molecules (and electrostatic interaction with the negatively charged DNA molecule is not expected), the high *f*_a _values indicate that the main type of interaction with the nucleic acid must be the binding to DNA grooves [28].

**Figure 8:**
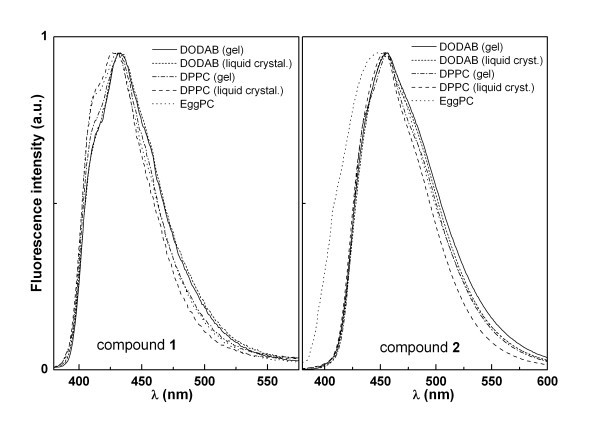
**Normalized fluorescence emission spectra of compounds 1 and 2 incorporated in liposomes of DPPC, Egg-PC and DODAB**.

Fluorescence experiments of both compounds encapsulated in liposomes of DPPC, DODAB and Egg-PC were carried out (Figure 8), in both gel (below *T*_m_) and liquid-crystalline (above *T*_m_) phases. The melting transition temperature of Egg-PC is very low [31] and this lipid is in the fluid liquid-crystalline phase at room temperature. Fluorescence spectra of compound **1 **incorporated in liposomes (Figure 8, Table 3) are roughly similar to the one obtained in pure water, regarding the band shape and maximum emission wavelength. Compound **2 **in liposomes presents emission spectra similar to those in polar solvents, significantly blue-shifted relative to water. In Egg-PC, a band enlargement is observed in the blue region, which can indicate two different locations of compound **2 **in these liposomes, one deep in the hydrophobic region and another more close to the lipid polar heads.

**Table 3:** Steady-state fluorescence anisotropy (*r*) values and maximum emission wavelengths (λ_em_) of compounds 1 and 2 incorporated in liposomes

	Compound 1		Compound 2	
	**λ**_**em**_**/nm**	*r*	**λ**_**em**_**/nm**	*r*
DPPC (25°C)	433	0.009	453	0.111
DPPC (55°C)	434	0.008	454	0.032
Egg-PC (25°C)	432	0.008	453	0.095
DODAB (25°C)	433	0.011	454	0.112
DODAB (55°C)	432	0.007	455	0.051
Glycerol (25°C)	437	0.166	472	0.202

Values in glycerol are also shown for comparison.

Fluorescence anisotropy (*r*) measurements (Table 3) can give relevant information about the location of the compounds in liposomes, as *r *increases with the rotational correlation time of the fluorescent molecule (and, thus, with the viscosity of the fluorophore environment) [26]. Anisotropy values in a viscous solvent (glycerol) were also determined, for comparison. Anisotropy results (Table 3) allow to conclude that compound **2 **is mainly located in the inner region of the lipid bilayer, feeling the penetration of some water molecules. The transition from the rigid gel phase to the liquid-crystalline phase is clearly detected by a significant decrease in anisotropy at 55°C observed in DPPC and DODAB liposomes. Compound **1 **exhibits a different behaviour and anisotropy is very low in all types of liposomes (and much lower than in glycerol, Table 3). Overall, the results indicate that compound **1 **prefers a hydrated and fluid environment and the transition from the gel phase to the liquid-crystalline phase is not detected. To further clarify the location of compound **1**, the solutions of liposomes with incorporated compound were passed through filters of 0.05 μm diameter. The fluorescence emission of the filtered solutions was negligible, indicating that compound **1 **is mainly in the liposome aqueous interior or located at the interfaces, with a very hydrated environment. This behaviour is similar to the observed previously for a benzothienopyridopyrimidone in lipid vesicles [27]. The encapsulation assays performed here may be important for future drug delivery applications of these potential antitumoral compounds using liposomes as drug carriers.

## Conclusions

The interaction with DNA of two new potential antitumoral fluorescent planar thieno[3,2-*b*]pyridine derivatives was studied using spectroscopic methods. Compound **2 **was shown to be the most intercalative compound in salmon sperm DNA (35%). The binding to DNA grooves seems to be the main type of interaction with the nucleic acid. Studies of incorporation of both compounds in liposomes of DPPC, Egg-PC and DODAB revealed that compound **2 **is mainly located in the hydrophobic region of the lipid bilayer, while compound **1 **prefers a hydrated and fluid environment. Our data thus suggest that both potential antitumoral compounds may be transported in liposomes for drug delivery applications.

## Abbreviations

DLS: dynamic light scattering; DODAB: dioctadecyldimethylammonium bromide; DPPC: dipalmitoyl phosphatidylcholine; DTS: Dispersion Technology Software; Egg-PC: egg yolk phosphatidylcholine; GUV: giant unilamellar vesicles; SV: small unilamellar vesicles.

## Competing interests

The authors declare that they have no competing interests.

## Authors' contributions

EMSC conceived the study, was responsible for its coordination and for the interpretation of results, and drafted the manuscript. MSDC carried out the liposome preparation and the fluorescence studies in liposomes. AROR carried out the experimental studies of the compounds interaction with DNA. RCC carried out the synthesis, purification and characterization of the new compounds. MJRPQ supervised the organic synthesis and participated in the draft of the manuscript. All authors read and approved the final manuscript.
